# Interventions for Bowen's disease: A systematic review and network meta‐analysis of randomized controlled trials

**DOI:** 10.1111/ddg.15866

**Published:** 2025-08-19

**Authors:** Yannick Foerster, Kristine Mayer, Marta Dechant, Julia Palaras, Tilo Biedermann, Oana‐Diana Persa

**Affiliations:** ^1^ Department of Dermatology TUM School of Medicine and Health Technical University of Munich Munich Germany

**Keywords:** Bowen, clearance rate, Squamous cell carcinoma in situ, therapy options

## Abstract

Bowen's disease (BD) is an intraepidermal malignancy that may progress to skin cancer, yet no study has compared interventions from randomized controlled trials (RCT). We synthesized RCT data to calculate lesion‐specific initial clearance rates, long‐term clearance rates, and cosmetic outcomes relative to placebo. We searched MEDLINE, EMBASE, CENTRAL and trial registers up to September 30, 2024, and included nine studies (672 patients, 844 lesions). Interventions compared to placebo and ranked via network meta‐analysis included surgery, imiquimod, photodynamic therapy (PDT), laser ablation (LA), laser‐assisted PDT (LA‐PDT), cryotherapy and 5‐fluoruracil (5‐FU). Bias was assessed using the revised Cochrane risk‐of‐bias tool. The study was registered in PROSPERO prior to data extraction (CRD42024583966).

All interventions showed higher lesion‐specific clearance rates than placebo. Regarding initial clearance, LA‐PDT ranked first, followed by surgery and LA. Surgery ranked first for long‐term clearance, followed by LA‐PDT and PDT. Cryotherapy and imiquimod ranked lowest for initial clearance and long‐term clearance. PDT, LA‐PDT, and 5‐FU showed the best cosmetic outcome, while surgery had the worst. Limitations included moderate to high bias and study heterogeneity.

Our findings support clinical decision‐making for BD, identifying LA‐PDT and PDT as effective options balancing efficacy and cosmetic results, and surgery as optimal for long‐term clearance.

## INTRODUCTION

Bowen's disease (BD), also known as cutaneous squamous cell carcinoma (cSCC) in situ, is a common type of non‐melanocytic intraepidermal malignancy. It appears as a well‐defined, slow‐growing, scaly patch on erythematous skin and is often misinterpreted as eczema or psoriasis.^1^ Most recent data from a nationwide cancer registry of the Netherlands demonstrated that the number of patients with BD doubled between 2005 and 2015.^2^ The disease peaks in the seventh decade of life and in many cases occurs on sun‐exposed areas,^2^ particularly the head and neck.^3^ The risk of progression to an invasive skin cancer is generally considered to be about 3%.^4^, ^5^ However, it remains unclear which lesions will become invasive. Therefore, early and consequent treatment is generally recommended by international guidelines.^6^, ^7^ Various topical and ablative interventions are available for the treatment of BD. This includes 5‐fluoruracil (5‐FU), imiquimod, photodynamic therapy (PDT), laser ablation (LA), laser‐assisted PDT (LA‐PDT), cryotherapy, and surgery. Unfortunately, the study landscape focusing on treatment options for BD is very limited. Most randomized controlled trials (RCTs) and available meta‐analyses primarily focus on head‐to‐head comparisons between PDT and other topical treatments, limiting the ability to directly compare different treatments.^8^, ^9^, ^10^, ^11^, ^12^, ^13^ Available reviews also included non‐randomized trials, which limits their reliability and generalizability for clinical decision‐making. Consequently, the comparative effects of available treatment modalities remain unclear, and current treatment recommendations are largely based on expert consensus. To address this gap, our study applies a network meta‐analysis (NMA), an advanced statistical approach that extends traditional meta‐analysis by allowing the simultaneous comparison of multiple interventions relative to one reference. Unlike conventional meta‐analysis, which only synthesizes direct comparisons from clinical trials, NMA enables the comparison of multiple interventions within a single analysis by integrating both direct data from trials (drug A vs. drug B) and indirect data across trials with shared comparators (drug A vs. drug C in study 1 and drug B vs. drug C in study 2, enabling an indirect comparison of A and B through C). This method maintains randomization and combines sample sizes from smaller studies to improve the statistical power of the analysis.^14^, ^15^, ^16^, ^17^ To provide the highest level of evidence, we only included RCTs. In our analysis, all treatment modalities for BD were compared to placebo as the reference and ranked with the P‐score method based on the NMA to provide guidance for clinical decision making.^18^ The analysis was performed for the primary outcomes initial complete clearance rate, long‐term complete clearance rate, and cosmetic result. As a secondary outcome, the absolute frequencies of adverse events for each treatment were evaluated.

## MATERIALS AND METHODS

### Protocol and registration

We registered our review in the *International Prospective Register of Systematic Reviews* (PROSPERO) prior to data extraction (CRD42024583966). We conducted our study in accordance with the *Preferred Reporting Items for Systematic Reviews and Meta‐Analyses* (PRISMA) including the NMA extension statement,^19^ the *Cochrane Handbook For Systematic Reviews* ^20^ and the *Enhancing Transparency in Reporting the Synthesis of Qualitative Research* (ENTREQ) statement.

### Eligibility criteria

Our eligibility criteria were defined in accordance with the population‐intervention‐comparison‐outcomes framework (PICO).^21^

*Population*: Patients with histopathological diagnosis of BD were included in our study.

*Intervention*: Patients had to be treated with one of the following treatment modalities: 5% 5‐FU, imiquimod, PDT, LA, LA‐PDT, cryotherapy, or surgery. For better comparability and to avoid unconnected and small subnetworks, PDT with aminolevulinate acid (ALA‐PDT) and methyl‐aminolevulinate (MAL‐PDT) as well as different light sources were pooled. Different ablative laser systems (Er:YAG and CO_2_ laser) were also pooled for the same reason. Sequential or combination therapies were excluded.

*Comparison*: All interventions were compared to placebo as the reference.

*Outcomes*: The following primary outcomes were investigated: *(1)* lesion‐specific initial clearance rate, reported from 1–3 months after treatment completion; *(2)* lesion‐specific long‐term clearance rate, reported from 6–12 months after treatment completion; and *(3)* cosmetic outcome. The secondary outcome was safety, which was defined as the proportion of patients experiencing any adverse event.

### Outcome measures

For BD, no standardized outcome measures for RCTs are available. We included only studies that reported lesion‐specific complete clearance rates. Outcomes *(1)* and *(2)* were dichotomous. Clearance rates were evaluated either clinically or through histopathological examination. For outcome *(3)*, numeric scales from 1–4 were reported. For better comparability, we considered 1–2 as poor/moderate and 3–4 as good/excellent. All effect sizes were compared to placebo as the reference and expressed as risk ratio (RR) with 95% confidence interval (95% CI). Due to inconsistent data, no estimated effect size could be calculated regarding the secondary outcome adverse events. Therefore, we summarized the absolute frequency of reported adverse events. Whenever possible, data were collected according to the intention‐to‐treat principle.

### Information sources and search strategy

We searched the electronic databases MEDLINE, EMBASE and the Cochrane Central Register of Controlled Trials (CENTRAL) from database inception to September 30, 2024. The search syntax is provided in Online Supplementary Table S1. In addition to the database search, the following clinical trial registers were manually searched for the keyword “Bowen” (last accessed September 30, 2024): EU Clinical Trials Register (https://www.clinicaltrialsregister.eu), Australian New Zealand Clinical Trials Registry (https://www.anzctr.org.au), US National Institute of Health Clinical Trial Register (https://clinicaltrials.gov).

### Study selection and data collection

Two investigators (YF and OP) independently used Ovid, a web‐based search platform, to screen all records by title and abstract, followed by full‐text review, against the eligibility criteria. The same authors also searched the clinical trial registers. Discrepancies were resolved by consensus. One study also included patients with actinic keratoses (AKs).^22^ Data for the subgroup of patients with BD was specifically extracted.

Only RCTs were considered for inclusion. No language restrictions were set. Both two‐arm and multi‐arm studies were included.

### Data synthesis

We conducted an NMA to give inferences regarding the relative effectiveness of multiple treatment options by using the frequentist NMA package netmeta (Version 3.1‐1) for RStudio (Version 4.4.2). We applied a random‐effects model due to the suspected heterogeneity across different studies.^14^ The R function pairwise was used to transform the arm‐based data set into a contrast‐based format, which is the correct format to conduct the NMA by using the netmeta package.^15^ Placebo was set as the reference. P‐scores were created by the netrank function.^18^ The p‐score is a statistical measure that indicates that one treatment is better than another, averaged over all competing treatments, and is based on point estimates and standard errors of the network estimates. Forest plots and network graphs were generated using the forest and netgraph functions.^23^ Between‐study heterogeneity was quantitatively analyzed with the I^2^ and τ^2^ statistics.^24^ To assess for inconsistency between direct and indirect evidence, we used the netsplit function to extract direct and indirect evidence from the network and compared both estimates using the node splitting method.^25^

### Risk of bias assessment and certainty of evidence

We assessed potential publication bias graphically using a funnel plot, which displays the centered effect estimates of different comparisons plotted against their standard errors. An asymmetric distribution of the funnel plot may suggest publication bias, which arises from the non‐publication of negative trials. Egger's test also proves statistical significance of funnel plot asymmetry.^26^ Two investigators (YF and OP) independently assessed the risk of bias for each outcome reported from the RCTs using the revised Cochrane risk‐of‐bias tool for randomized trials (RoB2).^27^ The tool provides a judgement of the overall bias arising from five different categories and generates colored risk of bias graphs: randomization process, deviations from the intended interventions, missing outcome data, measurement of the outcome and selection of the reported result. The certainty of evidence was evaluated using the Grading of Recommendations Assessment, Development, and Evaluation Working Group Guidance (GRADE) modified for pairwise meta‐analysis.^28^, ^29^ Conclusions from the NMA were drawn using the minimally contextualized framework approach.^30^

## RESULTS

### Study selection and study characteristics

**FIGURE 1:**
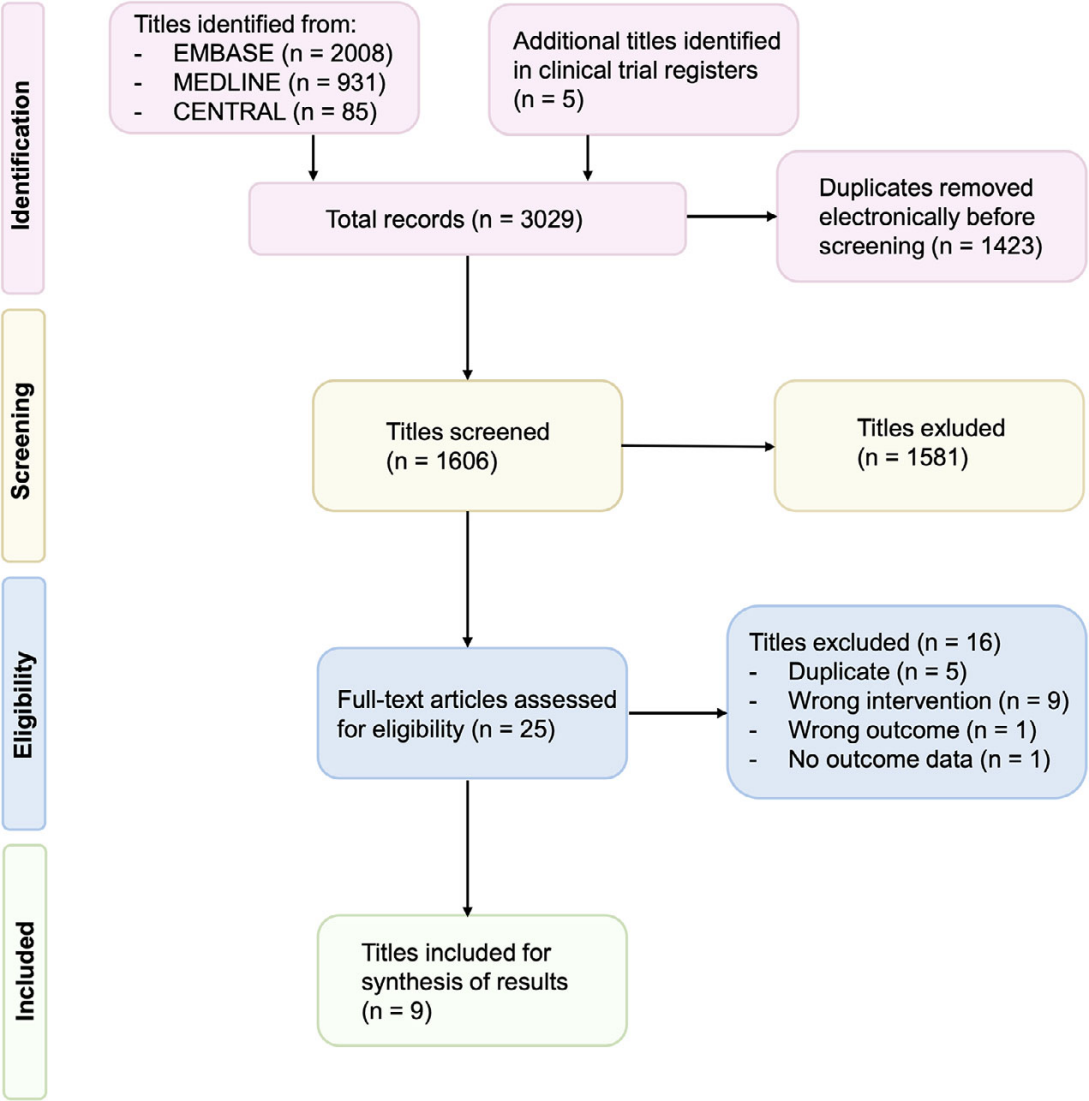
Flowchart of the selection process according to the Preferred Reporting Items for Systematic Reviews and Meta‐Analyses (PRISMA), including the extension statement for Network‐Metaanalysis (NMA).

Our systematic literature search identified 3,029 references. After automatically removing duplicates, 1,606 references remained. Of these, 1,581 studies were discarded after screening titles and abstracts. In total, 25 references underwent full‐text review, and nine studies, comprising 672 patients with 844 lesions, met the final inclusion criteria for data synthesis.^22^, ^31^, ^32^, ^33^, ^34^, ^35^, ^36^, ^37^, ^38^ The other studies were dismissed because they either investigated the wrong intervention outside of our scope (n  =  9), reported the wrong outcome (n  =  1) or had no outcome data available (n  =  1).^39^, ^40^, ^41^, ^42^, ^43^, ^44^, ^45^, ^46^ In addition, five more duplicates were identified and manually removed (Figure 1). The excluded studies with a brief description are listed in Online Supplementary Table S2.

All studies included immunocompetent patients, except for one study that focused only on post‐transplant disease.^22^ One study also included patients with AKs, so the data for BD had to be extracted manually.^22^ Out of the nine studies, seven were two‐armed,^22^, ^32^, ^33^, ^34^, ^36^, ^37^, ^38^ one was three‐armed,^31^ and one was four‐armed.^35^ All studies reported complete clearance rates within 1–3 months after treatment, which we defined as the initial clearance rate. Complete clearance rates measured 6–12 months after treatment were considered long‐term clearance rates. Of the nine studies, only four reported cosmetic outcomes.^22^, ^31^, ^34^, ^35^

Of the two‐armed studies, two studies each investigated the efficacy of PDT vs. LA‐PDT,^33^, ^34^ and two studies each examined PDT vs. 5‐FU.^22^, ^38^ The remaining two‐armed studies assessed LA vs. LA‐PDT,^32^ PDT vs. cryotherapy,^36^ and imiquimod  vs. placebo,^37^ respectively. One three‐armed study also investigated the efficacy of surgical excision compared to 5‐FU and PDT.^31^ The four‐armed study investigated cryotherapy, 5‐FU and PDT vs. placebo.^35^ A detailed overview of included studies is provided in Table 1.

**TABLE 1:** Overview of included studies.

					Sex									Outcomes reported					
Author	Year	Country	Patients	Mean age of patients (range)	Male (%)	Female (%)	Number of lesions	Diameter of the lesion (mm)	Intervention 1	Intervention 2	Intervention 3	Intervention 4	Duration of follow‐up (months)	Initial Clearance (months after end of treatment)	Long‐term clearance (months after end of treatment)	Cosmetic Outcome	AE	Sample size calculation (Power)	Comment
Ahmady et al.	2024	Netherlands	250	76 (51–94)	92 (36.8)	158 (63.2)	250	4–40 (range)	Surgery (5mm margin)	5% 5‐FU (2 x daily 4 weeks)	MAL‐PDT (2 cycles)	/	12	Yes (3)	Yes (12)	Yes	Yes	Yes (0.8)	
Cai et al.	2015	China	18	52 (35–72)	8 (44.4)	10 (55.6)	22	26 (mean)	CO2 ablative laser (up to 3 cycles depending on response)	CO2 ablative laser assisted ALA‐PDT (up to 3 cycles depending on response)	/	/	6	Yes (1)	Yes (6)	No	Yes	NR	
Kim et al.	2018	South Korea	60	72 (NB)	24 (40.0)	36 (60.0)	84	≥ 20 (range)	MAL‐PDT (2 cycles)	Erbium:YAG ablative fractional laser assisted MAL‐PDT (1 cycle)	/	/	60	Yes (3)	Yes (12)	No	Yes	NR	
Ko et al	2014	South Korea	21	69 (35–88)	10 (47.6)	11 (52.4)	58	NR	MAL‐PDT (2 cycles)	Erbium:YAG ablative fractional laser assisted MAL‐PDT (1 cycle)	/	/	12	Yes (3)	Yes (12)	Yes	Yes	NR	
Morton et al.	2006	United Kingdom	225	74 (39–99)	NR	NR	275	6–40 (range)	Placebo	Cryotherapy (1 cycle)	5% 5‐FU (1 x daily in first week followed by 2 x daily for 3 weeks)	MAL‐PDT (2 cycles)	12	Yes (3)	Yes (12)	Yes	Yes	Yes (0.9)	
Morton et al.	1996	United Kingdom	19	76 (62–88)	NR	NR	40	≤ 21 (range)	ALA‐PDT (up to 2 cycles depending on response)	Cryotherapy (up to 3 cycles depending on response)	/	/	12	Yes (2)	Yes (6)	No	No	NR	
Patel et al.	2006	United Kingdom	31	74 (54–86)	11 (35.5)	20 (64.5)	31	4.8–42.1 (range)	Placebo	5% Imiquimod (1 x daily 16 weeks)	/	/	9	Yes (3)	Yes (9)	No	No	Yes (0.9)	
Perrett et al.	2007	United Kingdom	8	59 (46–71)	6 (75.0)	2 (25.0)	18	NR	MAL‐PDT (2 cycles)	5% 5‐FU (2 x daily 3 weeks)	/	/	6	Yes (3)	Yes (6)	Yes	Yes	NR	Organ transplant recipients
Salim et al.	2003	United Kingdom	40	76 (65–88)	32 (80.0)	8 (20.0)	66	5–40 (range)	ALA‐PDT (up to 2 cycles depending on response)	5% 5‐FU (1 x daily in first week followed by 2 x daily for 3 weeks)	/	/	12	Yes (3)	Yes (12)	No	Yes	NR	

*Abbr*.: AE, adverse event; 5‐FU, 5‐fluoruracil; MAL‐PDT, methyl‐aminolevulinate photodynamic therapy; ALA‐PDT, aminolevulinate acid photdynamic therapy; NR, not reported

### Risk of bias assessment

**FIGURE 2:**
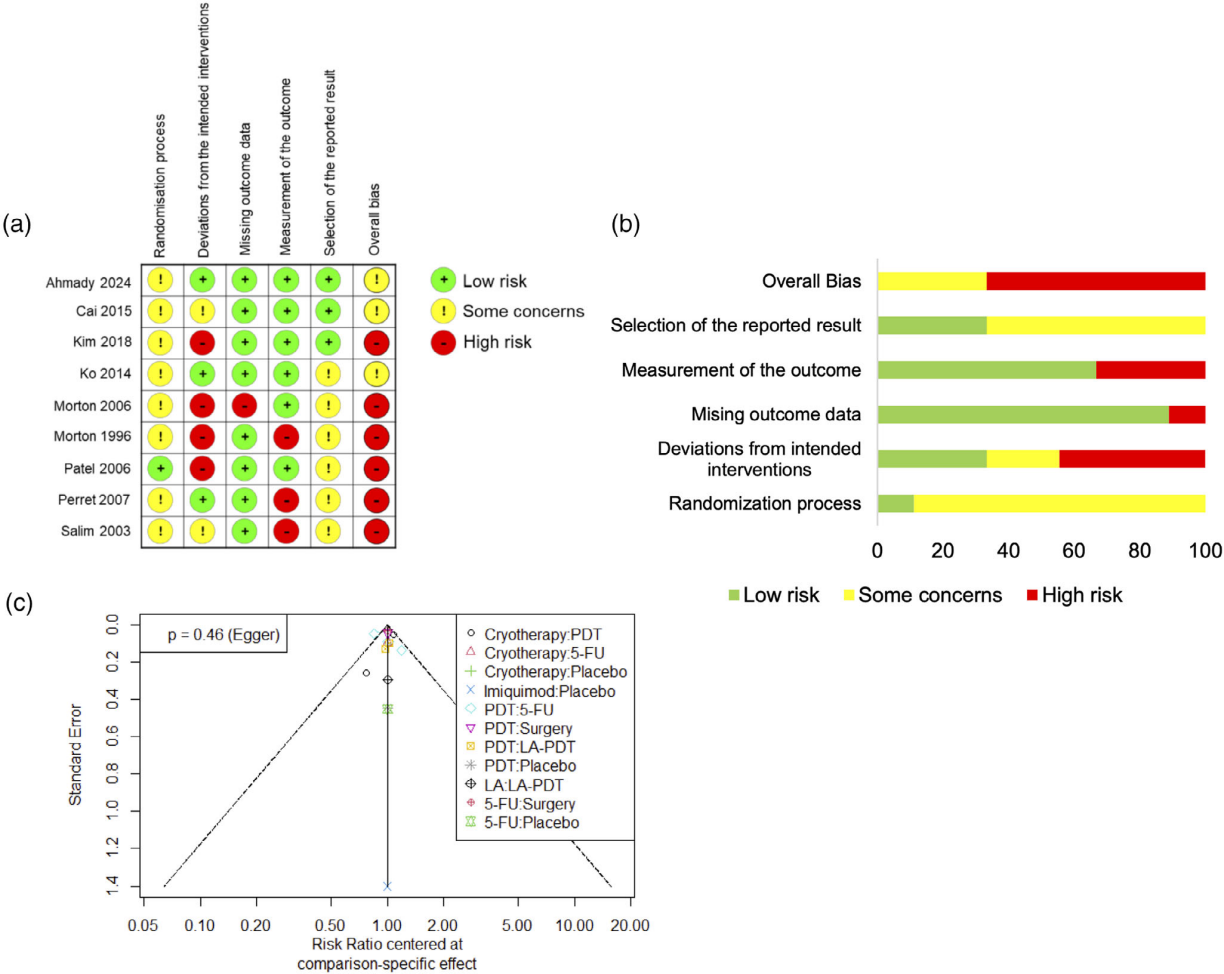
(a) Risk of bias for each outcome was evaluated by two independent authors according to the revised Cochrane risk‐of‐bias tool for randomized trials (RoB2). Risk of bias for each domain was stratified as low risk, some concerns or high risk. (b) The traffic light plot shows the proportion of studies judged as low risk, some concerns, or high risk of bias across the different domains. The figure shows risk of bias assessment for outcomes initial clearance and long‐term clearance. (c) Comparison‐adjusted funnel plot shows no signs of asymmetry, which may suggest potential publication bias.

Regarding the outcomes initial clearance and long‐term clearance, the overall risk of bias was rated moderate (some concerns) for three studies and high for the remaining six studies (Figure 2a,b). Only two studies provided information on how a random allocation sequence was achieved.^31^, ^33^ Only one study explained concealment of allocation.^37^ Conclusively, eight of nine studies raised some concerns regarding the overall randomization process according to RoB2, while only one study was rated with low concerns.^37^ Three studies raised low concerns about possible deviations from the intended treatment, indicating that both investigators and patients were adequately blinded and an appropriate analysis was used to estimate the effect of assignment to the intervention (intention‐to‐treat analysis).^22^, ^31^, ^34^ Of the remaining studies, two had some concerns^32^, ^38^ and four studies were at high risk.^33^, ^35^, ^36^, ^37^ Outcome was generally well reported. Only one study was at high risk for missing outcome data,^35^ while the remaining studies had a low risk. Regarding outcome measurement, six studies were considered low risk, and three were rated as high risk.^22^, ^36^, ^38^ Three studies were at low risk for the selection of the reported result,^31^, ^32^, ^33^ and the remaining six raised some concerns. Regarding cosmetic outcome, two studies showed a high risk of bias, and two studies showed a moderate risk of bias (Online Supplementary Figure S1).

Asymmetry in a comparison‐adjusted funnel plot, which may suggest potential publication bias, was neither visually apparent nor detected by Egger's test (p  =  0.46) (Figure 2c).

### Initial clearance

**FIGURE 3:**
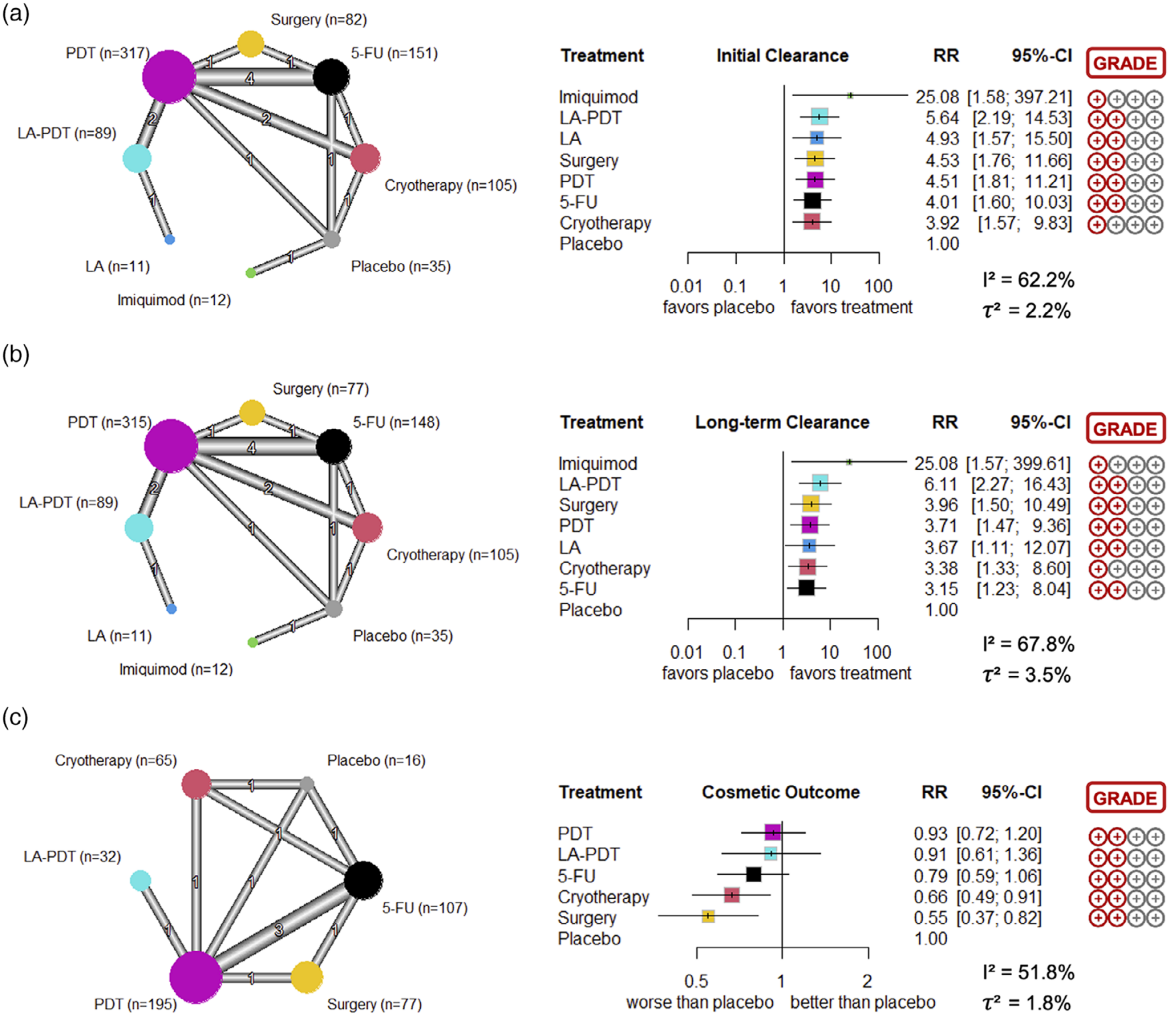
Network graphs and forest plots for each individual outcome: (a) lesion‐specific initial clearance rate (1‐3 months after treatment), (b) lesion‐specific long‐term clearance rate (6–12 months after treatment), and (c) cosmetic outcome. The risk ratio (RR) compared to placebo as the reference treatment was the main effect estimate. *Abbr*.: 95% CI, 95% confidence interval; LA‐PDT, laser‐assisted photodynamic therapy; LA, laser ablation; PDT, photodynamic therapy; 5‐FU, 5‐fluoruracil.

Data from nine studies were included in the NMA for lesion‐specific initial clearance. A total of 16 head‐to‐head comparisons provided direct evidence (Figure 3a). All treatments showed significant higher clearance rates compared to placebo. Imiquimod showed the highest clearance rates (RR, 25.08; 95% CI, 1.58–397.21), but the evidence is mainly based on one study that compared imiquimod to placebo.^37^ In this study, eleven out of fifteen patients treated with imiquimod achieved resolution of their BD, while none in the placebo group achieved resolution. In our NMA, imiquimod was followed by LA‐PDT (5.64; 2.19–14.53), LA (4.93; 1.57–15.50), surgery (4.53; 1.76–11.66), PDT (4.51; 1.81–11.21), and 5–FU (4.01; 1.60–10.03). Cryotherapy showed the least efficacy of all investigated treatment modalities (3.92; 1.57–9.83). Effect estimates of all direct and indirect comparisons for all primary outcomes are provided in Table 2. According to GRADE, the certainty of evidence was somewhat limited for all treatment modalities. Heterogeneity of the studies was moderate (I^2^  =  62.2%, τ^2^  =  2.2%).

**TABLE 2:** Overview of direct, indirect and combined evidence for each comparison based on the network meta‐analysis (NMA). Certainty of evidence was rated according to the Grading of Recommendations Assessment, Development, and Evaluation Working Group Guidance (GRADE), modified for pairwise meta‐analysis.

	DIRECT EVIDENCE			INDIRECT EVIDENCE		NMA	
Comparison	Number of Comparisons	RR [95% CI]	Certainty of Evidence	RR [95% CI]	Certainty of Evidence	RR [95% CI]	Certainty of Evidence
*Initial Clearance*							
5‐FU vs. Cryotherapy	1	0.96 [0.68; 1.36]	●●°°^2^	1.15 [0.70; 1.89]	●●°°	1.02 [0.77; 1.36]	●●°°
5‐FU vs. Imiquimod	0	NA		0.16 [0.01; 2.94]	●°°°	0.16 [0.01; 2.94]	●°°°
5‐FU vs. LA	0	NA		0.81 [0.40; 1.67]	●●°°	0.81 [0.40; 1.67]	●●°°
5‐FU vs. LA‐PDT	0	NA		0.71 [0.52; 0.98]	●●°°	0.71 [0.52; 0.98]	●●°°
5‐FU vs. PDT	4	0.90 [0.74; 1.09]	●●°°^2^	0.39 [0.07; 2.01]	●●●°	0.89 [0.73; 1.08]	●●●°
5‐FU vs. Placebo	1	3.93 [1.55; 9.99]	●●°°^2^	7.14 [0.05; 1092.89]	●●°°	4.01 [1.60; 10.03]	●●°°
5‐FU vs. Surgery	1	0.96 [0.71; 1.29]	●●●°^1^	0.55 [0.27; 1.12]	●●°°	0.88 [0.67; 1.16]	●●●°
Cryotherapy vs. Imiquimod	0	NA		0.16 [0.01; 2.88]	●°°°	0.16 [0.01; 2.88]	●°°°
Cryotherapy vs. LA	0	NA		0.80 [0.38; 1.67]	●●°°	0.80 [0.38; 1.67]	●●°°
Cryotherapy vs. LA‐PDT	0	NA		0.70 [0.48; 1.00]	●●°°	0.70 [0.48; 1.00]	●●°°
Cryotherapy vs. PDT	2	0.86 [0.66; 1.13]	●●°°^2^	0.95 [0.42; 2.16]	●●°°	0.87 [0.67; 1.13]	●●°°
Cryotherapy vs. Placebo	1	4.08 [1.62; 10.25]	●●°°^2^	0.01 [0.00; 942.29]	●●°°	3.92 [1.57; 9.83]	●°°°^4^
Cryotherapy vs. Surgery	0	NA		0.87 [0.60; 1.24]	●●°°	0.87 [0.60; 1.24]	●●°°
Imiquimod vs. LA	0	NA		5.08 [0.26; 101.13]	●°°°	5.08 [0.26; 101.13]	●°°°
Imiquimod vs. LA‐PDT	0	NA		4.45 [0.24; 82.50]	●°°°	4.45 [0.24; 82.50]	●°°°
Imiquimod vs. PDT	0	NA		5.56 [0.30; 102.03]	●°°°	5.56 [0.30; 102.03]	●°°°
Imiquimod vs. Placebo	1	25.08 [1.58; 397.21]	●°°°^2^, ^3^	NE		25.08 [1.58; 397.21]	●°°°
Imiquimod vs. Surgery	0	NA		5.53 [0.30; 102.49]	●°°°	5.53 [0.30; 102.49]	●°°°
LA vs. LA‐PDT	1	0.87 [0.46; 1.67]	●●°°^1^, ^3^	NE		0.87 [0.46; 1.67]	●●°°
LA vs. PDT	0	NA		1.09 [0.55; 2.19]	●●°°	1.09 [0.55; 2.19]	●●°°
LA vs. Placebo	0	NA		4.93 [1.57; 15.50]	●●°°	4.93 [1.57; 15.50]	●●°°
LA vs. Surgery	0	NA		1.09 [0.52; 2.29]	●●°°	1.09 [0.52; 2.29]	●●°°
LA‐PDT vs. PDT	2	1.25 [0.97; 1.62]	●●●°^1^	NE		1.25 [0.97; 1.62]	●●°°
LA‐PDT vs. Placebo	0	NA		5.64 [2.19; 14.53]	●●°°	5.64 [2.19; 14.53]	●●°°
LA‐PDT vs. Surgery	0	NA		1.24 [0.85; 1.81]	●●●°	1.24 [0.85; 1.81]	●●●°
PDT vs. Placebo	1	4.41 [1.76; 11.05]	●●°°^2^	16.69 [0.01; 19130.66]	●●°°	4.51 [1.81; 11.21]	●●°°
PDT vs. Surgery	1	0.91 [0.67; 1.23]	●●●°^1^	1.57 [0.79; 3.13]	●●°°	0.99 [0.75; 1.31]	●●●°
Surgery vs. Placebo	0	NA		4.53 [1.76; 11.66]	●●°°	4.53 [1.76; 11.66]	●●°°
*Long‐term Clearance*							
5‐FU vs. Cryotherapy	1	1.03 [0.66; 1.61]	●●°°^2^	0.84 [0.53; 1.32]	●●°°	0.93 [0.68; 1.28]	●●°°
5‐FU vs. Imiquimod	0	NA		0.13 [0.01; 2.33]	●°°°	0.13 [0.01; 2.33]	●°°°
5‐FU vs. LA	0	NA		0.86 [0.39; 1.89]	●●°°	0.86 [0.39; 1.89]	●●°°
5‐FU vs. LA‐PDT	0	NA		0.51 [0.34; 0.79]	●●°°	0.51 [0.34; 0.79]	●●°°
5‐FU vs. PDT	4	0.83 [0.65; 1.07]	●●°°^2^	1.41 [0.35; 6.60]	●●●°	0.85 [0.67; 1.08]	●●●°
5‐FU vs. Placebo	1	3.28 [1.25; 8.61]	●●°°^2^	1.63 [0.03; 79.74]	●●°°	3.15 [1.23; 8.04]	●●°°
5‐FU vs. Surgery	1	0.88 [0.62; 1.26]	●●●°^1^	0.44 [0.19; 1.04]	●●°°	0.79 [0.57; 1.10]	●●●°
Cryotherapy vs. Imiquimod	0	NA		0.13 [0.01; 2.50]	●°°°	0.13 [0.01; 2.50]	●°°°
Cryotherapy vs. LA	0	NA		0.92 [0.42; 2.04]	●●°°	0.92 [0.42; 2.04]	●●°°
Cryotherapy vs. LA‐PDT	0	NA		0.55 [0.36; 0.85]	●●°°	0.55 [0.36; 0.85]	●●°°
Cryotherapy vs. PDT	2	0.92 [0.71; 1.20]	●●°°^2^	0.76 [0.26; 2.24]	●●°°	0.91 [0.71; 1.17]	●●°°
Cryotherapy vs. Placebo	1	3.19 [1.24; 8.21]	●●°°^2^	26.25 [0.10; 6849.90]	●●°°	3.38 [1.33; 8.60]	●°°°^4^
Cryotherapy vs. Surgery	0	NA		0.85 [0.57; 1.28]	●●°°	0.85 [0.57; 1.28]	●●°°
Imiquimod vs. LA	0	NA		6.84 [0.34; 139.38]	●°°°	6.84 [0.34; 139.38]	●°°°
Imiquimod vs. LA‐PDT	0	NA		4.11 [0.22; 77.64]	●°°°	4.11 [0.22; 77.64]	●°°°
Imiquimod vs. PDT	0	NA		6.76 [0.36; 125.24]	●°°°	6.76 [0.36; 125.24]	●°°°
Imiquimod vs. Placebo	1	25.08 [1.57; 399.61]	●°°°^2^, ^3^	NE		25.08 [1.57; 399.61]	●°°°
Imiquimod vs. Surgery	0	NA		6.33 [0.34; 119.17]	●°°°	6.33 [0.34; 119.17]	●°°°
LA vs. LA‐PDT	1	0.60 [0.31; 1.17]	●●°°^1^, ^3^	NE		0.60 [0.31; 1.17]	●●°°
LA vs. PDT	0	NA		0.99 [0.47; 2.09]	●●°°	0.99 [0.47; 2.09]	●●°°
LA vs. Placebo	0	NA		3.67 [1.11; 12.07]	●●°°	3.67 [1.11; 12.07]	●●°°
LA vs. Surgery	0	NA		0.93 [0.41; 2.10]	●●°°	0.93 [0.41; 2.10]	●●°°
LA‐PDT vs. PDT	2	1.65 [1.16; 2.33]	●●●°^1^	NE		1.65 [1.16; 2.33]	●●°°
LA‐PDT vs. Placebo	0	NA		6.11 [2.27; 16.43]	●●°°	6.11 [2.27; 16.43]	●●°°
LA‐PDT vs. Surgery	0	NA		1.54 [0.96; 2.49]	●●●°	1.54 [0.96; 2.49]	●●●°
PDT vs. Placebo	1	3.81 [1.49; 9.75]	●●°°^2^	1.58 [0.01; 335.20]	●●°°	3.71 [1.47; 9.36]	●●°°
PDT vs. Surgery	1	0.84 [0.59; 1.21]	●●●°^1^	1.66 [0.72; 3.81]	●●°°	0.94 [0.67; 1.30]	●●●°
Surgery vs. Placebo	0	NA		3.96 [1.50; 10.49]	●●°°	3.96 [1.50; 10.49]	●●°°
*Cosmetic Outcome*							
5‐FU vs. Cryotherapy	1	1.16 [0.78; 1.71]	●●°°^2^	1.30 [0.67; 2.51]	●●°°	1.19 [0.85; 1.67]	●●°°
5‐FU vs. LA‐PDT	0	NA		0.87 [0.60; 1.26]	●●°°	0.87 [0.60; 1.26]	●●°°
5‐FU vs. PDT	3	0.85 [0.69; 1.06]	●●°°^2^	NE		0.85 [0.69; 1.06]	●●°°
5‐FU vs. Placebo	1	0.77 [0.54; 1.09]	●●°°^2^	0.84 [0.51; 1.40]	●●°°	0.79 [0.59; 1.06]	●●°°
5‐FU vs. Surgery	1	1.54 [1.08; 2.19]	●●●°^1^	0.76 [0.25; 2.31]	●●°°	1.44 [1.03; 2.02]	●●●°
Cryotherapy vs. LA‐PDT	0	NA		0.73 [0.47; 1.13]	●●°°	0.73 [0.47; 1.13]	●●°°
Cryotherapy vs. PDT	1	0.71 [0.51; 0.97]	●●°°^2^	0.85 [0.27; 2.71]	●●°°	0.72 [0.53; 0.97]	●●°°
Cryotherapy vs. Placebo	1	0.66 [0.49; 0.91]	●●°°^2^	NE		0.66 [0.49; 0.91]	●●°°
Cryotherapy vs. Surgery	0	NA		1.21 [0.78; 1.88]	●●°°	1.21 [0.78; 1.88]	●●°°
LA‐PDT vs. PDT	1	0.98 [0.72; 1.33]	●●●°^1^	NE		0.98 [0.72; 1.33]	●●●°
LA‐PDT vs. Placebo	0	NA		0.91 [0.61; 1.36]	●●°°	0.91 [0.61; 1.36]	●●°°
LA‐PDT vs. Surgery	0	NA		1.66 [1.05; 2.61]	●●●°	1.66 [1.05; 2.61]	●●●°
PDT vs. Placebo	1	0.94 [0.72; 1.23]	●●°°^2^	0.83 [0.37; 1.86]	●●°°	0.93 [0.72; 1.20]	●●°°
PDT vs. Surgery	1	1.59 [1.12; 2.25]	●●●°^1^	3.27 [1.04; 10.28]	●●°°	1.69 [1.21; 2.36]	●●●°
Surgery vs. Placebo	0	NA		0.55 [0.37; 0.82]	●●°°	0.55 [0.37; 0.82]	●●°°

^1^ Moderate risk of bias (‐1),

^2^ High risk of bias (‐2),

^3^ Imprecision (large confidence interval or small sample size) (‐1),

^4^ Incoherence between direct and indirect evidence (‐1). ●°°°, very low; ●●°°, low; ●●●°, moderate; ●●●●, high

*Abbr*.: RR, risk ratio; 95% CI, 95% confidence interval; NA, not applicable; NE, not estimable; LA‐PDT, laser‐assisted photodynamic therapy; LA, laser ablation; PDT, photodynamic therapy; 5‐FU, 5‐fluoruracil

### Long‐term clearance

Nine studies were analyzed for lesion‐specific long‐term clearance. This also included 16 direct head‐to‐head comparisons (Figure 3b). All treatments were significantly superior in achieving long‐term clearance compared to placebo, and imiquimod also showed the highest relative effect estimate (25.08; 1.57–399.61). Second highest RR was achieved by LA‐PDT (6.11; 2.27–16.43), followed by surgery (3.96; 1.50–10.49), PDT (3.71; 1.47–9.36), LA (3.67; 1.11–12.07), cryotherapy (3.38; 1.33–8.60), and 5‐FU (3.15; 1.23–8.04). Certainty of evidence was generally low, and heterogeneity was considered moderate (I^2^  =  67.8%, τ^2^  =  3.5%).

### Cosmetic outcome

Regarding cosmetic outcome, only four studies including eleven head‐to‐head comparisons were analyzed (Figure 3c). Cryotherapy (0.66; 0.49–0.91) and surgery (0.55; 0.37–0.82) showed significantly lower RR compared to placebo, indicating a worse cosmetic result. The remaining interventions were not statistically inferior to placebo (5‐FU: 0.79; 0.59–1.06; LA‐PDT: 0.91; 0.61–1.36; PDT: 0.93; 0.72–1.20). Certainty of evidence was low and heterogeneity moderate (I^2^  =  51.8%, τ^2^  =  1.8%).

### Treatment ranking

Treatments were ranked using a minimally contextualized framework (Table 3). LA‐PDT was ranked best for initial clearance, closely followed by surgery, LA, and PDT. Cryotherapy and imiquimod ranked last. Regarding long‐term clearance, surgery was ranked best, followed by LA‐PDT and PDT, while imiquimod and cryotherapy ranked last. PDT ranked best for cosmetic outcome followed by LA‐PDT and 5‐FU. Cryotherapy and surgery ranked last for cosmetic outcome.

**TABLE 3:** Ranking for each intervention in terms of lesion‐specific initial clearance rate (1‐3 months after treatment), lesion‐specific long‐term clearance rate (6–12 months after treatment), and cosmetic outcome. The risk ratio (RR) compared to placebo as the reference treatment was the main effect estimate. Treatment ranking is based on the minimally contextualized framework approach for network meta analyses, which considers effect estimates, the certainty of evidence, and treatment rankings according to their p‐scores.

Certainty of evidence, and classification of intervention	Rank	Intervention	RR [95% CI] compared to placebo	p‐score
*Lesion‐specific initial clearance (1–3 months after treatment)*				
Low certainty of evidence (⊕⊕◯◯)				
All interventions were superior to placebo	1	LA‐PDT	5.64 [2.19; 14.53]	0.8931
	2	Surgery	4.53 [1.76; 11.66]	0.8038
	3	LA	4.93 [1.57; 15.50]	0.7982
	4	PDT	4.51 [1.81; 11.21]	0.5792
	5	5‐FU	4.01 [1.60; 10.03]	0.3240
Very low certainty of evidence (⊕◯◯◯)				
All interventions were superior to placebo	6	Cryotherapy	3.92 [1.57; 9.83]	0.3417
	7	Imiquimod	25.08 [1.58; 397.21]	0.2562
*Lesion‐specific long‐term clearance (6–12m after treatment)*				
Low certainty of evidence (⊕⊕◯◯)				
All interventions were superior to placebo	1	Surgery	3.96 [1.50; 10.49]	0.9121
	2	LA‐PDT	6.11 [2.27; 16.43]	0.8940
	3	PDT	3.71 [1.47; 9.36]	0.5782
	4	LA	3.67 [1.11; 12.07]	0.4288
	5	5‐FU	3.15 [1.23; 8.04]	0.3190
Very low certainty of evidence (⊕◯◯◯)				
All interventions were superior to placebo	6	Imiquimod	25.08 [1.57; 399.61]	0.4910
	7	Cryotherapy	3.38 [1.33; 8.60]	0.3342
*Cosmetic outcome*				
Low certainty of evidence (⊕⊕◯◯)				
Category 1: Not inferior to placebo	1	PDT	0.93 [0.72; 1.20]	0.7440
	2	LA‐PDT	0.91 [0.61; 1.36]	0.6082
	3	5‐FU	0.79 [0.59; 1.06]	0.4078
Category 2: Inferior to placebo	4	Cryotherapy	0.66 [0.49; 0.91]	0.2089
	5	Surgery	0.55 [0.37; 0.82]	0.1813

*Abbr*.: LA‐PDT, laser‐assisted photodynamic therapy; LA, laser ablation; PDT, photodynamic therapy; 5‐FU, 5‐fluoruracil

### Adverse events

**FIGURE 4:**
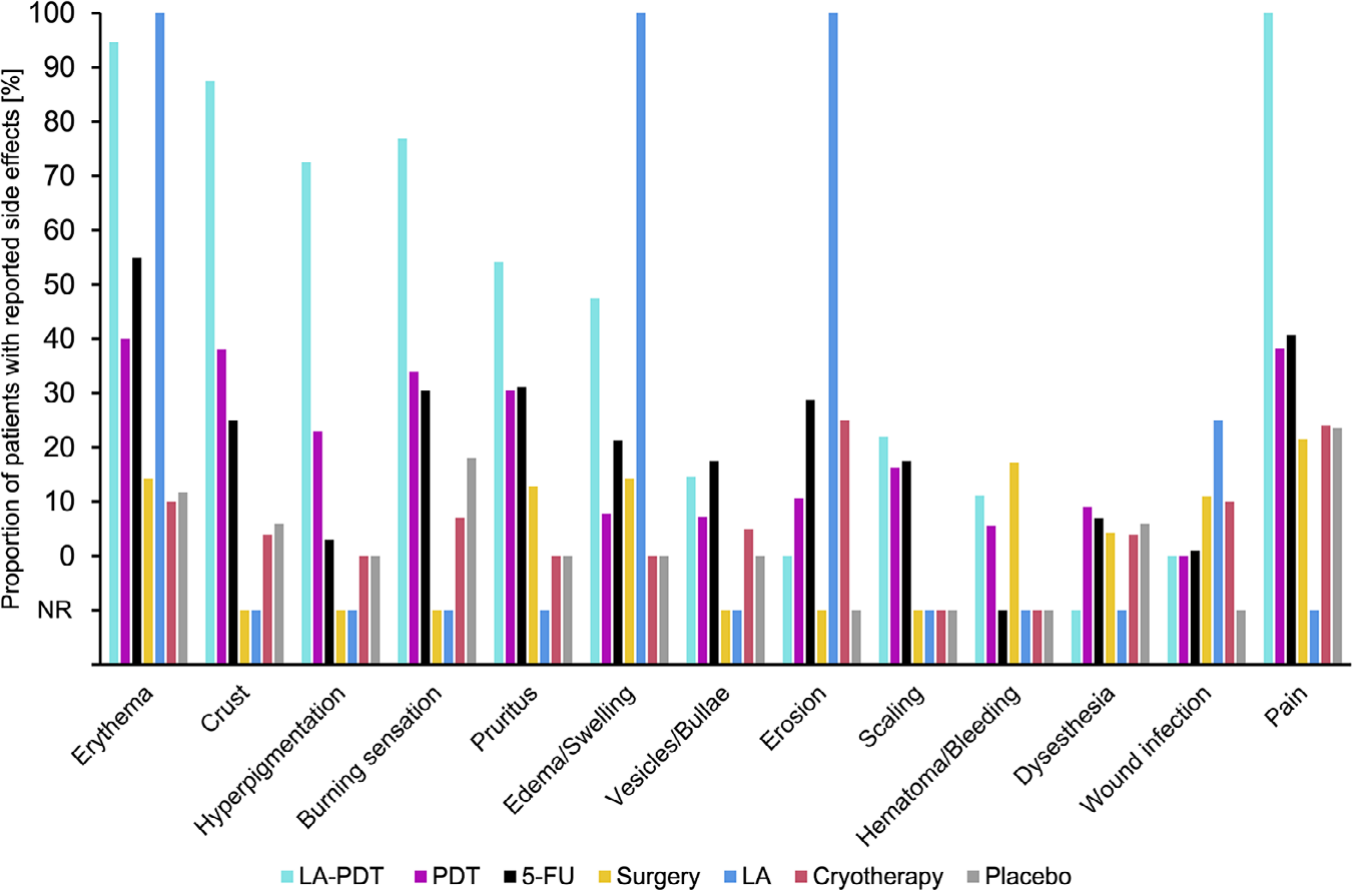
The grouped bar chart indicates the proportion of patients treated with any of the investigated interventions who experienced side effects. No side effects were reported for imiquimod. The averages were adjusted according to the group sizes of the individual studies. *Abbr*.: NR, not reported; LA‐PDT, laser‐assisted photodynamic therapy; LA, laser ablation; PDT, photodynamic therapy; 5‐FU, 5‐fluoruracil.

The incidence of adverse events was highly heterogeneous and inconsistently reported across the included studies, so data synthesis based on an NMA was not possible. An overview of the reported adverse events is provided in Figure 4. The averages were adjusted according to the group sizes of the individual studies (Online Supplementary Table S3).

## DISCUSSION

Our network meta‐analysis is the first to comprehensively evaluate the relative efficacy of multiple treatment modalities for BD that were investigated in RCTs, offering a comparative assessment based on both direct and indirect evidence. The analysis provides insights into lesion‐specific initial clearance, long‐term clearance, and cosmetic outcomes of various interventions. Overall, nine RCTs with a sample size of 672 patients and 844 lesions were included for data synthesis.

In terms of clearance rates, all treatments demonstrated significantly higher efficacy compared to placebo, with imiquimod showing the highest relative effect estimates for both initial clearance and long‐term clearance. However, it is important to note that the only direct evidence for imiquimod was derived from a single study with a small sample size, which demonstrated significant superiority of imiquimod over placebo. While the effect estimate for imiquimod should be interpreted with caution, multiple non‐randomized trials also report comparable response rates of up to 93%.^47^, ^48^, ^49^ LA‐PDT ranked first for initial clearance, followed by surgery, LA and PDT. This indicates that these interventions may provide more consistent and reliable outcomes for initial clearance of BD compared to topical treatments such as 5‐FU, cryotherapy, and imiquimod. This is in line with the findings of two meta‐analyses showing that PDT is more effective than 5‐FU and cryotherapy for the treatment of BD.^8^, ^9^ The systematic review by Xue et al.^8^ also showed that PDT combined with ablative fractional CO_2_ laser is superior to PDT. These findings strongly support the sequence of our treatment ranking, although we could not find any references directly comparing LA‐PDT with topicals such as 5‐FU, cryotherapy, or imiquimod. For long‐term clearance, similar trends were observed. While LA‐PDT demonstrated a higher RR, surgery ranked first for long‐term clearance. It is well‐known that surgery delivers excellent long‐term clearance rates.^50^ In contrast to other ablative or topical interventions, the lesion is typically removed with a safety margin or under micrographic control. Compared to surgery, other interventions carry a higher risk that a few malignant cells may evade treatment. This can lead to late recurrences, even if the lesion initially appeared to be cleared.

Cosmetic outcomes are a critical factor in the treatment of BD, particularly given that lesions often occur on visible areas like the face and neck. Our analysis found that cryotherapy and surgery were associated with poorer cosmetic outcomes compared to placebo. In contrast, treatments like PDT, LA‐PDT and 5‐FU showed more favorable cosmetic outcomes, making them attractive options for patients prioritizing both efficacy and cosmetic considerations.

The reporting of adverse events across the included studies was inconsistent, making it difficult to draw firm conclusions regarding the safety profiles of each treatment. However, based on the available data, no significant safety concerns emerged that would warrant the exclusion of any treatment modality from clinical practice. More standardized reporting on adverse events in future trials would greatly enhance the ability to assess the risk‐benefit ratio of each intervention.

One major strength of an NMA is the ability to integrate data from a broad range of studies, allowing a comparison of treatments that have not been directly tested against each other in head‐to‐head RCTs. However, the overall quality of evidence was limited by a small number of direct comparisons and heterogeneity across studies, which arises from differences in patient populations, study designs, interventions and outcomes. For example, there is no standardized outcome set available for BD as it is for AKs,^51^ so we focused on lesion‐specific complete clearance rates. Many of the included studies also had a high risk of bias, particularly in terms of the randomization process, which limited the certainty of evidence. Furthermore, only three of the included studies reported a transparent sample size and power calculation, which may affect the reliability and generalizability of the findings. Additionally, the time span of the included studies was quite long (1996–2024), introducing potential variability in study methodologies, diagnostic criteria, and analytical techniques over time. These factors may contribute to heterogeneity in the results and should be considered when interpreting the overall conclusions. Additional bias may arise from the fact that we had to combine ALA‐PDT and MAL‐PDT as well as different ablative laser systems for better comparability and to avoid small and unconnected subnetworks. One study also included immunosuppressed patients, which may also bias our results. The comparison‐adjusted funnel plot showed no signs of asymmetry, but the validity of the funnel plot and Egger's test is limited due to the small number of studies. However, considering the present study landscape, we could not identify any evidence indicating publication bias.

In conclusion, our NMA provides a comprehensive comparison of treatments for BD, offering evidence for clinical decision‐making. LA‐PDT and PDT emerge as strong candidates for balancing efficacy and cosmetic outcomes, while surgery remains the most reliable option for long‐term clearance despite its less favorable cosmetic profile. The findings underscore the need for more rigorous and standardized trials to better assess the long‐term efficacy, safety, and cosmetic outcomes of BD treatments.

## CONFLICT OF INTEREST STATEMENT

None.

## Supporting information



Supplementary information

Supplementary information
