# Supplementary material for: Interventions for Bowen's disease: A systematic review and network meta‐analysis of randomized controlled trials
Source: J Dtsch Dermatol Ges. 2025 Aug 19;23(11):1373–85. doi: 10.1111/ddg.15866 (PMC12619046; doi:10.1111/ddg.15866)
Supplement: Supplementary file 1 — Supplementary information [file DDG-23-1373-s002.docx]

FIGURE S1 (a) Risk of bias for each outcome was evaluated by two independent authors according to the revised Cochrane risk-of-bias tool for randomized trials (RoB2). Risk of bias for each domain was stratified as low risk, some concerns or high risk. (b) The traffic light plot shows the proportion of studies judged as low risk, some concerns, or high risk of bias across the different domains. The figure shows risk of bias assessment for cosmetic outcome.

TABLE S1 Search syntax. Electronic databases MEDLINE, EMBASE and the Cochrane Central Register of Controlled Trials (CENTRAL) were searched from database inception to September 30, 2024. Two investigators (YF and OP) independently used Ovid, a web-based search platform, to screen all records by title and abstract, followed by full-text review, against the eligibility criteria.

**Table S1**

| # | Searches |
| --- | --- |
| 1 | Morbus Bowen.mp. |
| 2 | bowen.mp. |
| 3 | Bowen's disease.mp. |
| 4 | squamous cell carcinoma in situ.mp. |
| 5 | laser.mp. or laser/ |
| 6 | PDT.mp. |
| 7 | photodynamic therapy.mp. |
| 8 | imiquimod.mp. |
| 9 | cryo.mp. |
| 10 | cryotherapy.mp. |
| 11 | cryosurgery.mp. |
| 12 | 5-FU.mp. |
| 13 | 5-fluoruracil.mp. |
| 14 | fluoruracil.mp. |
| 15 | mohs.mp. |
| 16 | 1 or 2 or 3 or 4 |
| 17 | 5 or 6 or 7 or 8 or 9 or 10 or 11 or 12 or 13 or 14 or 15 |
| 18 | 16 and 17 |

TABLE S2 Overview of studies excluded during the full-text review with brief description. ALA-PDT, aminolevulinate acid photdynamic therapy; MAL-PDT, methyl-aminolevulinate photodynamic therapy; 5-FU, 5-fluoruracil; BD, Bowen’s disease

**Table S2**

| Author | Interventions | Reason for exclusion | Description |
| --- | --- | --- | --- |
| de Haas 2007 | ALA-PDT single illumination vs. ALA-PDT two-fold illumination | Wrong intervention | We did not distinguish between single illumination and two-fold illumination to avoid small and unconnected subnetworks |
| Puizina-Ivic 2008 | ALA-PDT single illumination vs. ALA-PDT two-fold illumination | Wrong intervention | We did not distinguish between single illumination and two-fold illumination to avoid small and unconnected subnetworks |
| Morton 2000 | ALA-PDT red light vs. ALA-PDT green light | Wrong intervention | We did not distinguish between red and green light illumination to avoid small and unconnected subnetworks |
| Lui 2004 | Intravenous verteporfin with red light at 60 vs. 120 vs. 180 J/cm2 | Wrong intervention | We did not distinguish between different energy densities (fluence) to avoid small and unconnected subnetworks |
| Ellen 2007 | ALA-PDT single illumination vs. ALA-PDT two-fold illumination | Wrong intervention | We did not distinguish between single illumination and two-fold illumination to avoid small and unconnected subnetworks |
| Genouw 2018 | Fractional CO_2_ laser vs. continous CO2 laser | Wrong intervention | We did not distinguish between continous and fractional laser treatment to avoid small and unconnected subnetworks |
| Wu 2018 | ALA-PDT vs. ALA-PDT after Plum-blossom needling | Wrong intervention | We excluded Plum-blossom needling since it is not a well-established treatment for BD and to avoid small and unconnected subnetworks |
| Morton 2005 | MAL-PDT vs. Cryotherapy vs. 5-FU vs Placebo | Duplicate | The title was already included in our analysis (Morton 2006) |
| Morton 2004 | MAL-PDT vs. Cryotherapy vs. 5-FU vs Placebo | Duplicate | The title was already included in our analysis (Morton 2006) |
| Ibbotson 2022 | Low-irradiance ALA-PDT vs. Conventional irradiance ALA-PDT | Wrong intervention | We did not distinguish between low-irradiance and conventional irradiance ALA-PDT to avoid small and unconnected subnetworks |
| Mizutani 2012 | ALA-PDT with excimer-dye laser vs. PDT with metal-halide lamp | Wrong intervention | We did not distinguish between the source of irradiance ALA-PDT to avoid small and unconnected subnetworks |
| NCT00384124 | Topical Imiquimod vs. Placebo | Duplicate | The title was already included in our analysis (Patel 2006) |
| NCT03909646 | Surgery vs. MAL-PDT vs. 5-FU | Duplicate | The title was already included in our analysis (Ahmady 2024) |
| NCT03320447 | Er:YAG ablative fractional laser-assisted methyl aminolevulinate photodynamic therapy vs. MAL-PDT | Duplicate | The title was already included in our analysis (Kim 2018) |
| NCT03012009 | CO_2_ ablative fractional laser-assisted methyl aminolevulinate photodynamic therapy vs. MAL-PDT | No outcome data available | Study closed, last update posted 2018-01-25, no outcome data were available |
| 2005-005171-14 | MAL-PDT vs. ALA-PDT | Wrong outcome | Study was excluded due to wrong primary outcome (pain) |

*Abbr.:* PDT, photodynamic therapy; 5-FU, 5-fluoruracil; ALA-PDT, PDT with aminolevulinate acid; MAL-PDT, PDT with methyl-aminolevulinate

TABLE S3 Frequency of adverse events (AEs). LA-PDT, laser-assisted photodynamic therapy; PDT, photodynamic therapy; 5-FU, 5-fluoruracil; LA, laser ablation.

**Table S3**

| Treatment | Study | Adverse Event | | | | | | | | | | | | | | | | | | | | | | | | | | | | | | | | | | | | | | |
| --- | --- | --- | --- | --- | --- | --- | --- | --- | --- | --- | --- | --- | --- | --- | --- | --- | --- | --- | --- | --- | --- | --- | --- | --- | --- | --- | --- | --- | --- | --- | --- | --- | --- | --- | --- | --- | --- | --- | --- | --- |
|  |  | ***Erythema*** | | | ***Crust*** | | | ***Hyperpigmentation*** | | | ***Burning sensation*** | | | ***Pruritus*** | | | ***Edema/Swelling*** | | | ***Vesicles/Bullae*** | | | ***Erosion*** | | | ***Scaling*** | | | ***Hematoma/Bleeding*** | | | ***Dysesthesia*** | | | ***Wound infection*** | | | ***Pain*** | | |
|  |  | **AEs** | **Total** | **[%]** | **AEs** | **Total** | **[%]** | **AEs** | **Total** | **[%]** | **AEs** | **Total** | **[%]** | **AEs** | **Total** | **[%]** | **AEs** | **Total** | **[%]** | **AEs** | **Total** | **[%]** | **AEs** | **Total** | **[%]** | **AEs** | **Total** | **[%]** | **AEs** | **Total** | **[%]** | **AEs** | **Total** | **[%]** | **AEs** | **Total** | **[%]** | **AEs** | **Total** | **[%]** |
| LA-PDT | Kim 2018 | 28 | 30 | 93 | 24 | 30 | 80 | 23 | 30 | 77 | 22 | 30 | 73 | 21 | 30 | 70 | 9 | 30 | 30 | 3 | 30 | 10 |  |  |  |  |  |  |  |  |  |  |  |  |  |  |  |  |  |  |
|  | Cai 2015 | 10 | 10 | 100 |  |  |  |  |  |  |  |  |  |  |  |  | 10 | 10 | 100 |  |  |  |  |  |  |  |  |  |  |  |  |  |  |  |  |  |  |  |  |  |
|  | Ko 2014 | 17 | 18 | 94 | 18 | 18 | 100 | 12 | 18 | 67 | 15 | 18 | 83 | 5 | 18 | 28 |  |  |  | 4 | 18 | 22 | 0 | 18 | 0 | 4 | 18 | 22 | 2 | 18 | 11 |  |  |  | 0 | 18 | 0 | 18 | 18 | 100 |
|  | Total | 55 | 58 | 95 | 42 | 48 | 88 | 35 | 48 | 73 | 37 | 48 | 77 | 26 | 48 | 54 | 19 | 40 | 48 | 7 | 48 | 15 | 0 | 18 | 0 | 4 | 18 | 22 | 2 | 18 | 11 |  |  |  | 0 | 18 | 0 | 18 | 18 | 100 |
| PDT | Kim 2018 | 27 | 30 | 90 | 24 | 30 | 80 | 21 | 30 | 70 | 20 | 30 | 67 | 20 | 30 | 67 | 7 | 30 | 23 | 2 | 33 | 6 |  |  |  |  |  |  |  |  |  |  |  |  |  |  |  |  |  |  |
|  | Ahmady 2024 | 37 | 75 | 49 | 28 | 75 | 37 |  |  |  |  |  |  | 13 | 75 | 17 | 7 | 75 | 9 | 5 | 75 | 7 | 8 | 75 | 11 | 12 | 75 | 16 |  |  |  |  |  |  |  |  |  | 23 | 75 | 31 |
|  | Ko 2014 | 16 | 18 | 89 | 18 | 18 | 100 | 10 | 18 | 56 | 13 | 18 | 72 | 4 | 18 | 22 |  |  |  | 2 | 18 | 11 |  |  |  | 3 | 18 | 17 | 1 | 18 | 6 |  |  |  | 0 | 18 | 0 | 18 | 18 | 100 |
|  | Morton 2006 | 8 | 96 | 8 | 8 | 96 | 8 | 3 | 96 | 3 | 16 | 96 | 17 |  |  |  | 2 | 96 | 2 |  |  |  |  |  |  |  |  |  |  |  |  | 9 | 96 | 9 |  |  |  | 19 | 95 | 20 |
|  | Perrett 2007 |  |  |  | 8 | 8 | 100 | 1 | 8 | 13 |  |  |  | 3 | 8 | 38 |  |  |  |  |  |  |  |  |  |  |  |  |  |  |  |  |  |  |  |  |  | 8 | 8 | 100 |
|  | Salim 2003 |  |  |  |  |  |  |  |  |  |  |  |  |  |  |  |  |  |  |  |  |  |  |  |  |  |  |  |  |  |  |  |  |  |  |  |  | 14 | 19 | 74 |
|  | Morton 1996 |  |  |  |  |  |  |  |  |  |  |  |  |  |  |  |  |  |  |  |  |  |  |  |  |  |  |  |  |  |  |  |  |  |  |  |  |  |  |  |
|  | Total | 88 | 219 | 40 | 86 | 227 | 38 | 35 | 152 | 23 | 49 | 144 | 34 | 40 | 131 | 31 | 16 | 201 | 8 | 9 | 126 | 7 | 8 | 75 | 11 | 15 | 93 | 16 | 1 | 18 | 6 | 9 | 96 | 9 | 0 | 18 | 0 | 82 | 215 | 38 |
| 5-FU | Ahmady 2024 | 50 | 80 | 63 | 23 | 80 | 29 |  |  |  | 31 | 80 | 39 | 29 | 80 | 36 | 17 | 80 | 21 | 14 | 80 | 18 | 23 | 80 | 29 | 14 | 80 | 18 |  |  |  |  |  |  | 1 | 80 | 1 | 31 | 80 | 39 |
|  | Morton 2006 | 10 | 30 | 33 | 4 | 30 | 13 | 1 | 30 | 3 | 2 | 30 | 7 | 5 | 30 | 17 |  |  |  |  |  |  |  |  |  |  |  |  |  |  |  | 2 | 30 | 7 |  |  |  | 10 | 30 | 33 |
|  | Salim 2003 |  |  |  |  |  |  |  |  |  |  |  |  |  |  |  |  |  |  |  |  |  |  |  |  |  |  |  |  |  |  |  |  |  |  |  |  | 10 | 15 | 67 |
|  | Total | 60 | 110 | 55 | 27 | 110 | 25 | 1 | 30 | 3 | 33 | 110 | 30 | 34 | 110 | 31 | 17 | 80 | 21 | 14 | 80 | 18 | 23 | 80 | 29 | 14 | 80 | 18 |  |  |  | 2 | 30 | 7 | 1 | 80 | 1 | 51 | 125 | 41 |
| Surgery | Ahmady 2024 | 10 | 70 | 14 |  |  |  |  |  |  |  |  |  | 9 | 70 | 13 | 10 | 70 | 14 |  |  |  |  |  |  |  |  |  | 12 | 70 | 17 | 3 | 70 | 4 | 8 | 70 | 11 | 15 | 70 | 21 |
|  | Total | 10 | 70 | 14 |  |  |  |  |  |  |  |  |  | 9 | 70 | 13 | 10 | 70 | 14 |  |  |  |  |  |  |  |  |  | 12 | 70 | 17 | 3 | 70 | 4 | 8 | 70 | 11 | 15 | 70 | 21 |
| LA | Cai 2015 | 8 | 8 | 100 |  |  |  |  |  |  |  |  |  |  |  |  | 8 | 8 | 100 |  |  |  | 8 | 8 | 100 |  |  |  |  |  |  |  |  |  | 2 | 8 | 25 |  |  |  |
|  | Total | 8 | 8 | 100 |  |  |  |  |  |  |  |  |  |  |  |  | 8 | 8 | 100 |  |  |  | 8 | 8 | 100 |  |  |  |  |  |  |  |  |  | 2 | 8 | 25 |  |  |  |
| Placebo | Morton 2006 | 2 | 17 | 12 | 1 | 17 | 6 | 0 | 17 | 0 | 3 | 17 | 18 | 0 | 17 | 0 | 0 | 17 | 0 | 0 | 17 | 0 |  |  |  |  |  |  |  |  |  | 1 | 17 | 6 |  |  |  | 4 | 17 | 24 |
|  | Total | 2 | 17 | 12 | 1 | 17 | 6 | 0 | 17 | 0 | 3 | 17 | 18 | 0 | 17 | 0 | 0 | 17 | 0 | 0 | 17 | 0 |  |  |  |  |  |  |  |  |  | 1 | 17 | 6 |  |  |  | 4 | 17 | 24 |
| Cryotherapy | Morton 2006 | 8 | 82 | 10 | 3 | 82 | 4 | 0 | 82 | 0 | 6 | 82 | 7 | 0 | 82 | 0 | 0 | 82 | 0 | 4 | 82 | 5 |  |  |  |  |  |  |  |  |  | 3 | 82 | 4 |  |  |  | 20 | 82 | 24 |
|  | Morton 1996 |  |  |  |  |  |  |  |  |  |  |  |  |  |  |  |  |  |  |  |  |  | 5 | 20 | 25 |  |  |  |  |  |  |  |  |  | 2 | 20 | 10 |  |  |  |
|  | Total | 8 | 82 | 10 | 3 | 82 | 4 | 0 | 82 | 0 | 6 | 82 | 7 | 0 | 82 | 0 | 0 | 82 | 0 | 4 | 82 | 5 | 5 | 20 | 25 |  |  |  |  |  |  | 3 | 82 | 4 | 2 | 20 | 10 | 20 | 82 | 24 |

*Abbr.:* AEs, adverse events; PDT, photodynamic therapy; LA-PDT, laser-assisted photodynamic therapy; 5-FU, 5-fluoruracil
